# The role of fenofibrate in the treatment of COVID-19

**DOI:** 10.1016/j.amsu.2021.102974

**Published:** 2021-11-03

**Authors:** Farah Yasmin, Muhammad Hamayl Zeeshan, Irfan Ullah

**Affiliations:** a Department of Internal Medicine, Dow Medical College, Dow University of Health Sciences, Karachi, Pakistan; b Department of Community Medicine, Kabir Medical College (Gandhara University), Peshawar, Pakistan

**Keywords:** COVID-19, Fenofibrate

## Abstract

In December 2019, a severe pneumonia-like illness emerged in the city of Wuhan, China which inevitably led to the Coronavirus disease-19 (COVID-19) pandemic caused by the SARS-CoV-2 virus. Due to the increase in infectivity and mortality caused by the virus, multiple therapeutic regimens are being suggested in order to help tackle this problem. The infectivity of SARS-CoV2 virus is due to its ability to attach itself to the ACE II receptors on the host cells via its viral spike protein (S protein) and inducing its entry into the target cell. The effects of the drug, Fenofibrate on the ACE II receptors and also, how the pharmaceutical properties of this drug can help prevent viral entry and eventually, reduce severity in COVID-19 patients. Since this drug has a good profile and is relatively safe to use, the risk-to-benefit analysis is positive and can be beneficial for patients infected with severe SARS-CoV2 infection.

## 1 Introduction

In December 2019, a severe pneumonia-like illness emerged in the city of Wuhan, China which inevitably led to the Coronavirus disease-19 (COVID-19) pandemic. The disease is known to be caused by severe acute respiratory syndrome coronavirus 2 (SARS-CoV-2) and owning to its rapid transmission, it was declared a pandemic by the World Health Organization (WHO) in March 2020 [1]. The SARS-CoV-2, formerly known as novel coronavirus (2019 n-COV), is a positive single-stranded RNA virus belonging to the family coronaviridae [2]. As of August 22^nd^ 2021, the virus has led to more than a total of 212,000,000 confirmed cases with more than 4,000,000 deaths worldwide [3]. Throughout the pandemic, many different treatments have been suggested which includes the use of drugs such as Remdesivir, Hydroxychloroquine, anti-viral, amongst others [4]. Another emerging drug which is being highlighted for the treatment of COVID-19 is Fenofibrate, belonging to a group of drugs known as fibrates.

Fenofibrate is a synthetic derivative of fibric acid; a peroxisome proliferator-activated receptor alpha (PPAR-a) agonist. The therapeutic effect of this drug is based on the mechanism that activation of the PPAR-a receptor leads to lipolysis due to an increase in activity of lipoprotein lipase [5]. The drug induces high-density lipoprotein (HDL) synthesis and also decreases the production of apolipoprotein C. As a result, there is an overall reduction in the serum level of triglycerides and low-density lipoprotein (LDL) cholesterol making it a potential therapeutic drug for hypertriglyceridemia and dyslipidemia. Fenofibrate belongs to the class of drug called fibrates, which helps in the reduction of serum triglyceride levels by inducing fatty acid oxidation via acyl CoA synthetase. Fenofibrate has anti-inflammatory, anti-oxidant and anti-angiogenic effects as well. They help to reduce tumor necrosis factor alpha (TNF-alpha), oxidative stress, Interleukin-6 (IL-6), vascular endothelial growth factor-1 (VEGF) signaling and are also effective in lowering fibrinogen levels [6]. Studies have also shown that potentially, fenofibrate is an effective therapeutic drug for patients with severe COVID-19 and especially for patients suffering from cytokine storm, a severe immune reaction in which excessive cytokines are released into the body that can induce pulmonary and several extra pulmonary complications including myocarditis, acute kidney injury and gastrointestinal manifestations (such as diarrhea and vomiting) [7].

## 2 The possible therapeutic effect of fenofibrate in reducing COVID-19 severity

SARS-Cov-2 is a positive sense single stranded RNA virus which is encapsulated by the spike (S) glycoproteins. These S spike proteins are responsible for the entry of the virus as the Receptor Binding Motifs (RBM) of the spike proteins interact with the host cell receptor, angiotensin converting enzyme II (ACE II) receptor, and mediates entry of the virus inside the host cell [8]. The ACE II receptors are located in numerous cells throughout the human body but these receptors are abundantly expressed in the epithelial lining of the pneumocyte cells of the respiratory system. Hence, these cellular sites are more prone to infections from the SARS-CoV-2 virus. The extrapulmonary tissues where the ACE II receptors are also expressed include the heart, kidney, endothelium and the luminal surface enterocytes of the small intestine [8,9]. Studies have been made which shows that ACE II is actually a dimer and multiple spike RBMs of the virus might interact with each ACE II dimer [10] inducing its entry into the host cell. Transmembrane protease serine 2 (TMPRSS2) is also another host cell enzyme that can facilitate entry of the virus via priming with the SARS-CoV-2 spike proteins [8].

At the site of the lungs during infection, the virus causes an over stimulation of the T-cells which leads to an excessive release of inflammatory mediators like TNF-a, IL-6, IL-8 and VEGF. As a result, these can further lead to complications like severe pneumonia, pulmonary edema, acute respiratory distress syndrome (ARDS) and also, death [11]. This is the main cause of mortality in patients with SARS-CoV-2 infection.

A recent study conducted by Davies SP et al., 2021 [12] under laboratory conditions investigated the effects of fenofibrate, along with other fibrates, in trying to combat the SARS-CoV-2 infection. It was based on the hypothesis that the extent of ACE II dimerization might affect its binding with the RBMs of the spike protein of SARS-CoV-2. In previous other studies, it was observed that dimerization of other receptors like endothelial growth factor (EGF) or fibroblast growth factor (FGF) receptors resulted in their endocytosis (internalization) [13,14]. Another study showed receptor internalization was also seen in monomeric and dimeric growth hormone receptors [15]. This theory of ACE II dimerization and viral infection was then tested out.

In this study [12], ELISA was performed in which recombinant ACE II were used to determine the effects of Fenofibrate and other fibrates on RBM-ACE II binding inhibition. In the result, the fibrate drugs that were used indicated a significant effect in the inhibition of the binding. Other than that, the DSF (Differential scanning fluorimetry) showed destabilization of the viral spike protein because of these drugs, in which Feno fibric acid had the most potent effect. When the drug is administered orally, it undergoes a reaction by carboxylesterases which converts the ester prodrug into the free acid, fenofibric acid. The fenofibric acid is shown to be more potent in the dimerization assay suggesting that the prodrug fenofibrate, which is the isopropyl ester of fenofibric acid, was comparatively inactive in inducing dimerization. This suggests that the free carboxylic acid form is necessary for this process to take place.

**Fig. 1:**
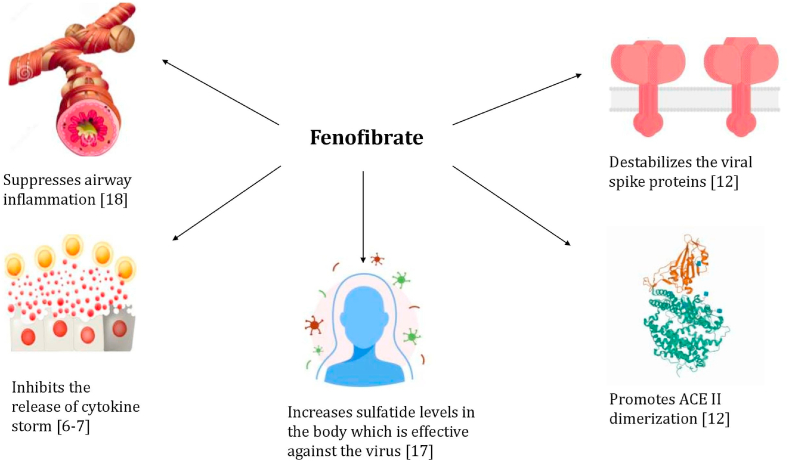
**Therapeutic effects of Fenofibrate against COVID-19**.

In conclusion of his study, it was observed that under different settings in the laboratory, fenofibrate and fenofibric acid both reduced viral infection by up to 70% [12]. Thus, the drug can become a useful and potential therapeutic agent in the treatment of severe COVID-19 infected patients. Further clinical trials are to be performed by the team in order to test this drug out in hospitalized COVID-19 patients. Another study conducted by Buschard et al. [16] recognized that a rise in sulfatide levels due to intake of fenofibrate can also help in the reduction of SARS-CoV-2 infection. Fenofibrate has been known to suppress airway inflammation as well, meaning the drug can also provide relief to patients suffering from severe respiratory symptoms as a result of SARS-CoV-2 infection as shown in Fig. 1 [17].

## 3 Benefits of fenofibrate as a COVID-19 medication

The drug is given orally, has lower costs and is easily available worldwide, it can be quite beneficial especially for low middle-income countries (LMIC). Even though vaccination programmes have been beneficial for preventing the spread of COVID-19, the availability of the vaccine and uptake rates are inconsistent for everyone [18]. Individuals, who cannot take the vaccine such as the elderly, patients under immunosuppressants, and patients with hyper immune disorders etc. can be administered fenofibrate for reducing COVID-19 symptoms and severity. The pharmacological properties of fenofibrate can also be considered for their effects in improving symptoms among COVID-19 patients. The pleiotropic effects of fenofibrate are also useful in preventing severity of the COVID-19 infection, they help to decrease the release of cytokines caused by the virus.

Furthermore, fenofibrate can be suitable drug of choice because it has a good safety profile. The drug does not possess any serious adverse effects, the more common ones are headache, dizziness and gastrointestinal side effects like nausea, vomiting or diarrhea. Only rarely, serious adverse reactions like hepatotoxicity or myopathy are seen when given alongside statins [19].

The drug is contraindicated in patients with any pre-existing liver disease, kidney disease, patients having a hypersensitive reaction to the drug and also to those who are breastfeeding. An appropriate risk-benefit analysis is recommended before administering fenofibrate to patients suffering from COVID-19 but after recent studies and ongoing clinical trials, it is likely that fenofibrate might become an effective agent for the treatment against the disease.

## Ethics approval and consent to participate

Not applicable.

## Consent for publication

Not applicable.

## Availability of data and materials

Data sharing is not applicable to this article as no datasets were generated or analyzed during the current study.

## Funding

None.

## Authors' contributions

FY: Conception of the study, drafting of the work, final approval, and agreeing to the accuracy of the work.

MHZ: Conception of the study, drafting of the work, final approval, and agreeing to the accuracy of the work.

IU: Supervision and critical revision of the manuscript, editing, final approval, and agreeing to the accuracy of the work.

## Funding sources

None.

## Declaration of competing interest

The authors declare that they have no competing interests.
